# Mepolizumab for hypereosinophilic syndrome: effectiveness and safety from real-world evidence

**DOI:** 10.3389/fimmu.2025.1704077

**Published:** 2025-11-14

**Authors:** Elvira Mora, María Laura Fox, Angelina Lemes, Beatriz Velasco, Jesús María Hernández-Rivas

**Affiliations:** 1 Hospital Universitario y Politécnico La Fe, Instituto de Investigación Sanitaria La Fe, Valencia, Spain; 2 Department of Hematology, Vall d’Hebron University Hospital, Experimental Hematology, Vall d’Hebron Institute of Oncology (VHIO), Barcelona, Spain; 3 Department of Medicine, Universidad Autónoma de Barcelona (UAB), Barcelona, Spain; 4 Department of Hematology, University Hospital of Gran Canaria Doctor Negrín, Las Palmas de Gran Canaria, Spain; 5 Specialty Care Medical Department, GSK, Madrid, Spain; 6 Department of Medicine, University of Salamanca, Salamanca, Spain; 7 Molecular Genetics in Oncohematology, Institute of Biomedical Research of Salamanca (IBSAL) - Cancer Research Center of Salamanca (USAL-CSIC), Salamanca, Spain

**Keywords:** mepolizumab, hypereosinophilic syndrome, antibodies, monoclonal, humanized, interleukin-5, treatment outcome

## Abstract

Hypereosinophilic syndrome (HES) is a rare condition characterized by elevated eosinophil levels and related symptoms of eosinophil-mediated organ damage. We reviewed the effectiveness and safety of mepolizumab for the treatment of HES. A scoping review was conducted following the PRISMA Scoping Reviews Checklist to identify real-world evidence of mepolizumab use in HES. In total, 36 references were identified as relevant and selected for review. Overall, 105 patients previously treated with glucocorticoids received mepolizumab at different dosages (range: 100–750 mg), routes of administration (subcutaneous/intravenous), and schedules (every 2–12 weeks). Remission rates were 57.1–76.0%. Most studies reported a range of 71.4–99.1% reduction in mean blood eosinophil counts with mepolizumab treatment. In addition, a glucocorticoid-sparing effect was observed; 85.7% of patients discontinued glucocorticoids after 12 months of mepolizumab administration. Mepolizumab was considered safe and well-tolerated and severe adverse events were rare. Mepolizumab provided clinically significant benefits in patients with HES in a real-world setting.

## 1 Introduction

**Figure 1:**
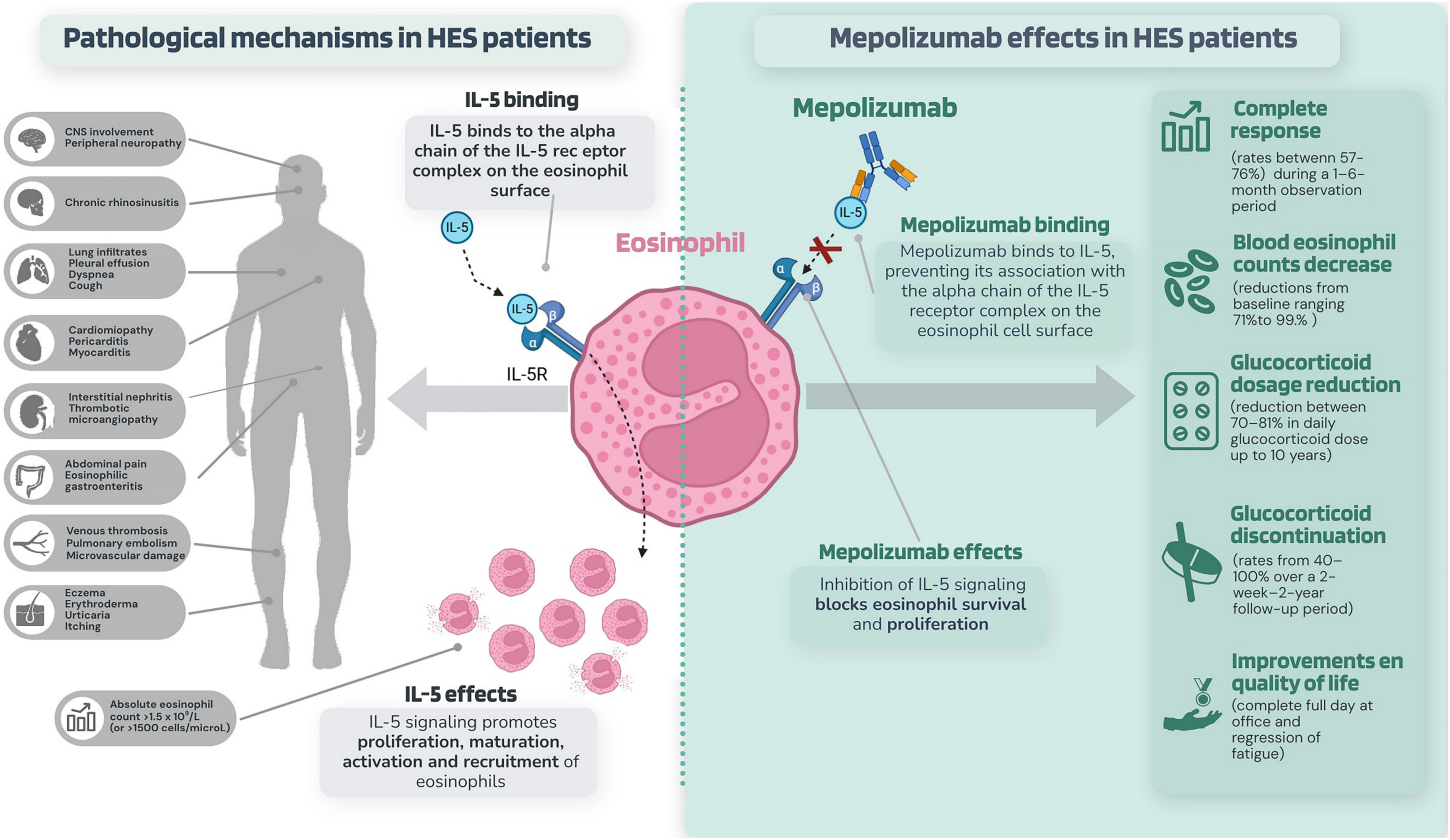
Pathological mechanisms in idiopathic and T lymphocytic variants of HES and the effects of mepolizumab.

Hypereosinophilic syndrome (HES) is a group of rare disorders characterized by elevated eosinophil levels in blood and/or tissues, associated with eosinophil-mediated organ damage or dysfunction (1, 2). Eosinophil activation can lead to tissue damage through various mechanisms, including the secretion of cytokines and granule products (e.g., major basic protein, eosinophil-derived neurotoxin), as well as the generation of lipid mediators (e.g., sulfidopeptide leukotrienes, platelet-activating factor, and granulocyte-macrophage colony-stimulating factor [GM-CSF]; **Figure 1**). The disease course and clinical manifestations are highly variable, with some patients experiencing persistent or progressive disease, while others have fluctuating disease activity with episodic worsening of symptoms (3). The most prevalent symptoms at initial presentation manifest with cutaneous (estimated to affect 37% of patients), gastrointestinal, and pulmonary involvement. However, at the time of diagnosis, other life-threatening complications, such as cardiovascular and neurological manifestations, have been reported in 5% and 4% of patients, respectively (3). The identification of HES poses significant challenges due to the necessity of excluding other eosinophilic disorders that present with similar symptoms (4).

The International Cooperative Working Group on Eosinophil Disorders includes the following criteria for the diagnosis of HES: a) blood eosinophilia of >1500 eosinophils/μL on two examinations with a minimum time interval of four weeks (this time limit is not required for cases with rapid onset eosinophil-related organ dysfunction); b) organ damage and/or dysfunction due to tissue eosinophilia; and c) exclusion of other disorders or conditions as the major reason for organ damage (5). Various classification systems have been proposed for HES. Thus, HES can be categorized according to clinical phenotype: myeloproliferative (M-HES), lymphocytic (L-HES), overlap, associated, or familial (6). M-HES is characterized by the clonal expansion of eosinophils in a primary myeloid neoplasm and accounts for approximately 10–20% of HES cases. The interstitial deletion in chromosome 4, resulting in the *FIP1L1*-*PDGFRA* fusion gene, is among the mutations associated with M-HES. This fusion gene causes autonomous proliferation of hematopoietic stem cells, increasing eosinophil counts in >80% of cases (7). Cytogenetic disturbances involving tyrosine kinases have been demonstrated as the likely source of other instances of M-HES, including various *PDGFRA*, *PDGFRB*, *FGFR1*, and *JAK2* gene fusions, as well as *JAK2* point mutations (6, 7). In L-HES cases, elevated eosinophil counts are caused by the overproduction of eosinophilopoietic cytokines by immunophenotypically aberrant T-cell populations (8). These interleukin-5 (IL-5)-producing T cells may or may not be clonal and exhibit, in most cases, a CD3^−^CD4^+^ phenotype (9).

The overall therapy objectives for patients with HES include the reduction of the absolute eosinophil count (AEC), amelioration of signs and symptoms, and prevention of disease progression (10), while minimizing therapy complications. Except for patients with a secondary cause of HES, for whom treatment should be targeted at the underlying disease, glucocorticoids remain the primary therapeutic approach for the treatment of most forms of HES, as well as severe and potentially life-threatening manifestations of HES in acute situations (11). For patients diagnosed with HES exhibiting an insufficient response to glucocorticoids or demonstrating intolerance to glucocorticoids, additional therapeutic options are recommended. These options may include imatinib (especially for subtypes associated with gene fusions involving *PDGFRA* or *PDGFRB* (12) in which imatinib should be the first line of treatment), immunomodulatory agents (such as interferon alpha, ciclosporin, or azathioprine), cytoreductive therapy (hydroxycarbamide), or monoclonal antibody therapy (mepolizumab and others) (13). The selection of treatment is dependent upon several factors, such as the type of HES, severity (cardiac, central nervous system, or thrombotic involvement), clinical course (continuous progression or recurrent episodes), and the patient’s individual characteristics (age, potential comorbidities) (14). Traditional treatments, such as glucocorticoids and cytotoxic and immunomodulatory drugs, exhibit variable efficacy and significant side effects (15). Patients diagnosed with early HES have a high initial response rate when treated with glucocorticoids as first-line monotherapy, with up to 85% of patients showing a positive response within one month of treatment. Nevertheless, a considerable percentage of these patients experience substantial adverse effects attributed to glucocorticoids or treatment resistance, with lack of efficacy being the most prevalent cause of treatment discontinuation (3). Given the prominent involvement of IL-5 as the primary cytokine responsible for promoting the survival and persistence of eosinophils in the etiology of HES (16), it has been suggested that therapies aimed at inhibiting eosinophils may have the potential to result in favorable therapeutic effects.

Treatments targeting IL-5/IL-5 receptor signaling include mepolizumab, benralizumab and reslizumab. Of these, the most widely investigated is mepolizumab, a humanized monoclonal antibody that binds to and neutralizes IL-5 (**Figure 1**) (17). Mepolizumab is the only biologic drug approved by the European Medicines Agency (EMA) and the United States Food and Drug Administration (FDA) (17, 18). The FDA approval includes adult and pediatric patients aged 12 years and older with HES for ≥6 months without an identifiable non-hematologic secondary cause (17). The EMA also approved mepolizumab in 2021 as an add-on treatment for adults with inadequately controlled HES without an identifiable non-hematologic secondary cause (18).

However, while it is true that the use of drugs such as mepolizumab provides the possibility of more effective and less toxic approaches to the treatment of HES, there is little available real-world data to guide their use in HES, and its long-term results have not yet been determined (15). Since the approval of mepolizumab, the experience with the drug has been reported as a few prospective and retrospective studies and isolated clinical cases. Due to the increase in the real-world use of mepolizumab, the demographic composition of patients with access to the treatment becomes more varied compared with those enrolled in randomized controlled trials (RCTs). Moreover, the stringent eligibility criteria employed in RCTs often exclude patients with respiratory comorbidities and other characteristics commonly observed in the real-world population with HES. Real-world evidence has provided insights into many areas of concern regarding the treatment of patients with HES, including potential long-term effects associated with mepolizumab administration. It is, therefore, beneficial to assess outcomes associated with mepolizumab use in everyday clinical practice. Here, we conducted a scoping review of the literature to assess the real-world effectiveness and safety of mepolizumab in patients with HES.

## 2 Methods

The Preferred Reporting Items for Systematic Reviews and Meta-Analyses (PRISMA) Extension for Scoping Reviews (PRISMA-ScR) checklist (19) and the methods given by Arksey and O’Malley (20) were used to guide this study. Five sequential methods were followed: (i) identifying the research question, (ii) identifying relevant studies, (iii) selecting eligible studies, (iv) charting the data, and (v) collating and summarizing the results. A primary investigator conducted the literature search, screening, review, and data charting. The study selection was conducted by an experienced investigator and the results were validated independently by all the authors. Local ethics committee approval was not required because this study was based on published data.

### 2.1 Identifying the research question

The primary research question was based on the Population, Intervention, Comparison, Outcomes, and Study (PICOs) framework: “What is the effectiveness and safety of mepolizumab in the treatment of patients with HES, as supported by real-world evidence?”. This question referred to several clinical outcomes, including clinical remission, blood eosinophil count, glucocorticoid maintenance dosage, and drug safety.

### 2.2 Identifying relevant studies

The review was designed to identify publications reporting data on the effectiveness and safety of mepolizumab in patients with HES in a real-world setting. Thorough electronic searches of the two main biological databases – Ovid Medline and EMBASE – were conducted; other literature sources such as conference proceedings, trial registries and other non-indexed reports were excluded from the search. The search strategies were adapted for each database, using a combination of free-text terms and medical subject headings (**Supplementary Materials**). The review included articles published in the database from its inception until May 19, 2023. Further searches for more relevant studies were carried out by manually examining the reference lists of the selected research papers and review articles. The search strategies were restricted to studies conducted on humans, with no limitations on language or publication year.

### 2.3 Study selection

Full-text articles and titles/abstracts were screened for inclusion based on the following eligibility criteria: [1] enrolled adult or pediatric patients with HES diagnosis according to validated criteria, irrespective of clinical stage or disease duration; [2] treatment with mepolizumab at any dose or route of administration; and [3] reported data from real-world evidence, including prospective and retrospective cohorts, cross-sectional and case-control studies, case series and case reports. Studies could have been published in any format, such as full papers or conference abstracts, but must have provided sufficient data to estimate outcomes. The exclusion criteria were as follows: [1] studies that included mixed populations; [2] studies that examined the effects of medications other than mepolizumab; [3] studies with any other design (e.g., clinical trials, narrative reviews, editorial comments, and letters).

### 2.4 Charting data and reporting the results

Data from publications meeting the eligibility criteria were collected by a single investigator using a data extraction template. Information gathered from each publication included: author(s), year of publication, study location, title, follow-up period, study population, intervention type, outcome measures, and critical results on the effectiveness and safety of mepolizumab. All data were entered and verified using a specifically designed ‘data form’ using the Excel database program. In order to address the risk of duplicate patient reporting, we cross-checked study author lists, institutions, and recruitment periods. When multiple papers pertaining to the same sample or research were published, preference was given to the most recent or comprehensive report. The results of our review were summarized qualitatively, and no quantitative analyses were planned. For all critical outcomes of interest, results have been summarized and presented separately for case series (prospective and retrospective) and case reports.

## 3 Results

### 3.1 Search results

**Figure 2:**
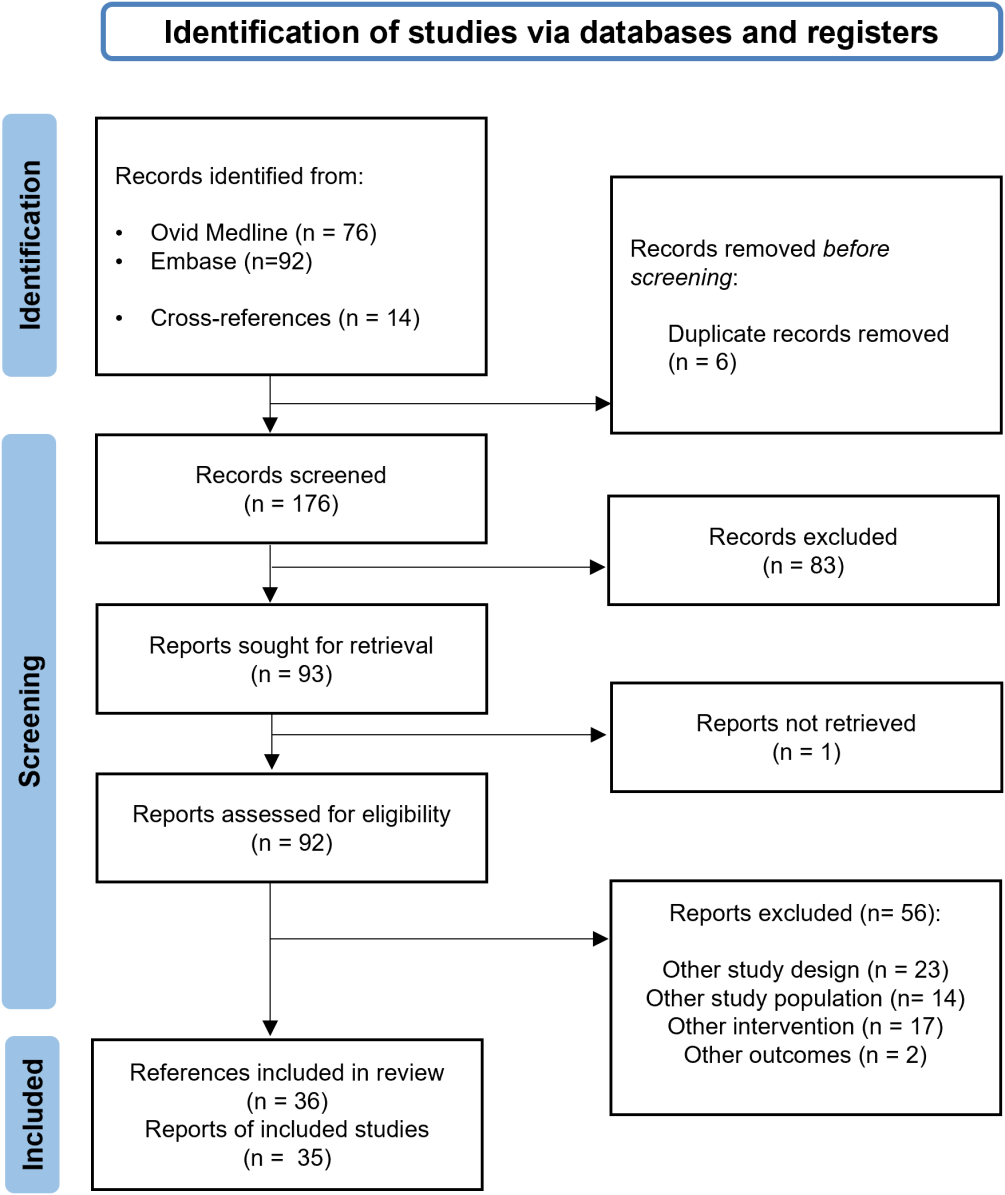
PRISMA flowchart with the main stages of the review process.

The electronic database searches identified 168 potentially eligible publications or abstracts. A manual search using the bibliography of select articles identified 14 records. After excluding 6 duplicate references and 83 publications or abstracts that did not meet the eligibility criteria (and were considered irrelevant), 93 full texts were retrieved to confirm their eligibility. Of the available references, it was not possible to access the complete text of one document, and a total of 56 entries were excluded. The main reasons for exclusion were study design (i.e., clinical trial) and intervention (i.e., medications other than mepolizumab). A total of 36 papers or abstracts that met the inclusion criteria were included in this review. Among selected studies, we combined 2 publications based on the same research (21, 22). We finally included 36 references covering 35 original studies. The PRISMA study flow diagram is illustrated in **Figure 2**.

### 3.2 Characteristics of included studies

Of the 35 studies that examined the effectiveness and safety of mepolizumab in real-world settings in 105 patients with HES, 2 (5.7%) were prospective series (23, 24), 5 (14.2%) were retrospective series (3, 9, 21, 25, 26), and 28 (80%) were case reports (8, 15, 27–52).

Most publications (65.7%) consisted of journal articles; 14.2% were reported as conference abstracts, and 20% were editorial letters. Publication dates ranged from 2003 to 2023, with more than a third of studies (13/35, 37.1%) published since 2018. Most studies were conducted in the United States (n=18, 51.4%), and Europe (n=10, 28.6%, predominantly Italy [n=4, 11.4%] and France [n=2, 5.7%]), while some patients were reported from Asia (n=5, 14.3%) and Oceania (n=1, 3.4%). Studies were conducted within a single center (including hospitals and clinics), except for one study involving patients from several centers in America and Europe (3).

### 3.3 Baseline characteristics of patients

The 35 unique study populations included a total of 105 patients diagnosed with HES. Most participants were derived from retrospective series (57.1%; n=60), while prospective and case reports contributed 13.3% (n=14) and 29.5% of the total (n=31), respectively. The number of patients per study ranged from 1 to 35 (21). Seven studies included pediatric patients (8 patients in total) (30, 31, 45, 47, 50–52). The mean/median age of recruited patients across the studies ranged from 3 (47) to 82 years (27), with the majority of patients in their 40s or 50s. Among the 24 studies that reported follow-up duration, the average follow-up duration ranged from 1 week (51) to 60 months (40), with follow-up periods most commonly spanning 1 to 12 months (**Table 1**). Among the participants who had accessible gender data, 46 (61.3%) were female. The study cohort comprised 42.5% of individuals diagnosed with idiopathic HES, 17.8% with L-HES, and 3.4% with M-HES. Other diagnoses such as hypereosinophilic undifferentiated syndrome (HEUS), Gleich syndrome, and overlap of various subtypes of HES were observed less frequently, accounting for 5.5%, 1.4%, and 28.1% of participants, respectively; 1.4% of participants had other organ-specific diagnoses. The median time from initial presentation to HES diagnosis ranged from 8 months (25) to 7.1 years (9). Most patients (96.4%) tested for the FIP1L1-PDGFRA fusion were negative (25, 26). In terms of organ involvement, cutaneous manifestation was the most common finding both in pediatric and adult populations, accounting for 62% and 50.5% of cases, respectively. Other manifestations observed in adult participants were respiratory (44.3%), constitutional (30.9%), and gastrointestinal symptoms (27.8%), while in children, the respiratory and constitutional manifestations were present in 37.5% of participants. Involvement of the cardiovascular and neurologic systems was reported in 15.5% and 12.4% of adults, respectively, and in 12.5% of pediatric participants (for both systems). A total of 23 studies documented median and mean baseline blood eosinophil counts, ranging between 0.85 (33) and 56.9×10^9^/L (29) among adult participants, with a median peak eosinophil count of 8.1×10^9^/L (interquartile range [IQR]: 3.81–20.0); mean baseline blood eosinophil counts in the pediatric subpopulation ranged between 1.5×10^9^/L (45) and 83.3×10^9^/L (50).

**Table 1:** Characteristics of study populations.

Author, year	Study location	Follow-up duration (months)	Number of patients	HES variant	Sex	Age, yrs	Eosinophil count (×10^9^/L) or (%)
Prospective series							
Maule, 2023 (23)	Italy	12	7	Idiopathic HES	NR	NR	NR
Paolini, 2010 (24)	Italy	26	7	NR	NR	NR	NR
Retrospective series							
Ogbobu, 2009 (3)	Multicentre	1	15	L-HES	NR	Median 45	6.6 (range 1.5–400)
Kuang, 2016 and 2018 (21, 22)	USA	6	35	20 Idiopathic; 3 M-HES; 6 L-HES; 6 Overlap;	22F 13M	Median 44	NR
Carpentier, 2020 (9)	India	22.3	5/26 treated with mepolizumab	24 L-HES 2 HEUS	5F	Median 43.5	3.81 (range 0.6–53.0)
Salomon, 2022 (25)	France	NR	1/20 treated with mepolizumab	Idiopathic HES	NR	Median 74	3.8
Benjamin, 2018 (26)	USA	6	4	NR	4F	Mean 49.5	NR
Case reports							
Plotz, 2003 (27)	USA	5	3	NR	3F	60–82	15–41%
Koury, 2003 (28)	USA	NR	1	NR	M	51	2–8
Wagner, 2009 (29)	USA	NR	1	M-HES	F	56	Up to 56.9
Mehr, 2009 (30)	Australia	18	1	HEUS	M	**9**	19.7
Schwartz, 2010 (41)	USA	24	3	NR	3M	47–65	NR
Kersey-Barrett, 2012 (32)	USA	20	1	Idiopathic HES	F	58	Up to 18
Bleeker, 2012 (33)	USA	21	1	Idiopathic HES	F	48	0.85
D’Elbée, 2013 (34)	France	NR	1	L-HES	M	65	41
Roufosse, 2015 (35)	Belgium	6	1	L-HES (Gleich syndrome)	F	49	NR
Klion, 2015 (36)	USA	NR	1	Idiopathic HES	F	42	8.9
Patel, 2016 (37)	USA	NR	1	NR	F	25	NR
Song, 2017 (38)	USA	7	1	Idiopathic HES	M	60	7.8 (0–0.5)
Brunet, 2018 (39)	USA	NR	1	M-HES	M	70	8.1
Matucci, 2018 (40)	Italy	60	1	Gleich syndrome	M	37	4.6
Schwarz, 2018 (41)	Germany	48	2	L-HES	1F 1M	**11–14**	Up to 45%
Mulvey, 2018 (42)	USA	3	1	Overlap HES	M	31	1.4
Klion, 2018 (15)	USA	NR	1	Idiopathic HES	F	50	NR
To, 2018 (43)	Japan	3	1	Chronic eosinophilic pneumonia	M	65	NR
Eng, 2020 (44)	USA	NR	1	L-HES	F	58	12.3
Domany, 2020 (45)	Israel	8	1	NR	M	**8**	1.5
Kurosawa, 2020 (46)	Japan	11	1	Idiopathic HES	F	56	NR
Weiss, 2021 (47)	Austria	NR	1	Idiopathic HES (suggestive of Wells syndrome)	F	**3**	47%
Helbig, 2021 (8)	USA	8	1	Idiopathic HES	F	58	>20
Di Nora, 2021 (48)	Italy	18	1	Eosinophilic myocarditis and Loeffler endocarditis	M	34	NR
Jonakowski, 2021 (49)	Poland	12	1	Idiopathic HES	M	59	>1.5
Eubanks, 2022 (50)	USA	2 weeks	1	NR	M	**6**	83.3
Cascio, 2022 (51)	USA	1 week	1	L-HES	M	**4**	68.6
Jue, 2022 (52)	Korea	NR	1	L-HES	F	**4**	NR

F, female; HES, Hypereosinophilic syndrome; HEUS, Hypereosinophilia of undetermined significance; L-HES, T lymphocytic variants of HES; M, male; M-HES, Myeloproliferative variants of HES; NR, Not reported. Ages below 18 years are shown in bold.

Thirty-five studies reported information on oral glucocorticoid use at baseline, with 100% of patients using glucocorticoids prior to mepolizumab treatment. Glucocorticoids, mainly prednisone and prednisolone, were prescribed with mean daily doses in the range of 2–80 mg/day. The median duration of glucocorticoid monotherapy was 55 months (range 14–174 months) in a retrospective series (9). The duration of glucocorticoid treatment varied in the case reports, ranging from 3 days (38, 46) to 20 years (31), and was mainly used for maintenance purposes. According to available data from 29 studies, 57 patients (54.2%) used previous treatments with additional agents prior to receiving mepolizumab, with imatinib (19.3%), hydroxyurea (15.8%), and alpha interferon (14%) being the most commonly used. **Table 1** summarizes the study characteristics and clinical characteristics at baseline.

### 3.4 Mepolizumab: effectiveness

The main indication for mepolizumab treatment across all the studies was the presence of glucocorticoid-refractory or immunosuppressive-refractory disease (n=29/38, 76.3%). However, alternative indications were reported, including the identification of potential issues related to the toxicity of glucocorticoids or interferon (IFN)-alpha (n=2, 5.3%) (30, 33), disability to taper glucocorticoids further (n=1, 2.6%) (51), the occurrence of life-threatening HES (n=1, 2.6%) (21). Five studies (13.2%) failed to provide information regarding the indication for mepolizumab (3, 9, 26, 48, 50). All 35 studies used mepolizumab at different dosages and routes of administration, in combination with or without glucocorticoids or other immunosuppressive drugs. Most participants received intravenous mepolizumab at 700–750 mg (n=77, 73.3%), while 22.8% (n=24) received 100 mg (n=21, 20%) or 300 mg (n=3, 2.8%) subcutaneously (SC). Only one case report analyzed the effect of 10 mg/kg intravenous mepolizumab infusions every 4 weeks in a pediatric patient (30). The administration frequency of mepolizumab also varied among the participants in the different studies. A significant proportion of participants (n=98, 93.3%) received mepolizumab every 4–6 weeks. However, a smaller number of people received mepolizumab at other intervals, including every week (n=1, 1%) (34), every 2 weeks (n=2, 1.9%) (28), or every 8–12 weeks (n=1, 1%) (31). A summary of the primary effectiveness findings is provided in **Table 2**.

**Table 2:** Summary of effectiveness and safety data from included real-world evidence.

Author, year	Mepolizumab dosages	Main effectiveness findings	Main safety findings
Prospective series			
Maule, 2023 (23)	100 mg Q4W	At 12 months: 6 (85.7%) had complete withdrawal of prednisone Drastic reduction in eosinophilic count (median 0.04×10^9^/L, p = 0.008)	No disease flares or adverse events
Paolini, 2010 (24))	750 mg Q4W	At 26 months: 5 (71.4%) had response 4 (57.1%) had symptom remission 2 (33.3%) had drastic reduction in eosinophil count	No mepolizumab-related adverse events nor allergic reactions during infusion
Retrospective series			
Ogbobu, 2009 (3)	750 mg Q4W	At 1 month: 12 (80%) had complete response with mepolizumab monotherapy (34 [75.5%] with mepolizumab in combination with glucocorticoids) 5 (11.1%) had partial response	Deaths (n = 4) Deaths secondary to eosinophilic heart disease (n = 2) Anti–IL-5 therapy discontinuation (n = 29): • lack of efficacy (n = 10) • medication intolerance (n = 1)
Kuang, 2016 and 2018 (21, 22)	750 mg Q4W	At 3 months: 20 (57.1%) had complete response 7 (20%) had partial response 20 (57.1%) had reduction in absolute eosinophil count within normal limits	At 5 years: Malignant neoplasms (n = 4) Deaths in mepolizumab group (n = 2)
Carpentier, 2020 (9)	700–750 mg Q4W	4 (80%) had clear-cut haematological response 2 (4%) had partial response 2 (4%) had progressive clinical worsening	1 (20%) had malignant neoplasm (lymphoma) 3 (60%) had lack of efficacy
Salomon, 2022 (25)	700 mg Q4W	1 (9.1%) had full symptomatic control	NR
Benjamin, 2018 (26)	100 mg Q4W	At 6 months: 4 (100%) had improvement in disease control Reduction in daily prednisone dose from 13.8 to 3.5 mg (p = 0.13) Reduction in oral glucocorticoids course from 1.5 to 0.3 (p = 0.13) Reduction in mean eosinophil count from 3.375 to 0.075×10^9^/L (p = 0.13)	NR
Case reports			
Plotz, 2003 (27)	750 mg, interval dosing NR	Decrease in blood eosinophil count (n=3) Improvement of symptoms (skin lesions, pruritus) within first weeks of mepolizumab initiation (n=3) Complete clinical remission (n=2) Symptoms worsening after mepolizumab discontinuation (n=1) and five weeks after second dose of mepolizumab (n=1)	No adverse events
Koury, 2003 (28)	750 mg Q2W	Transient partial improvement of pulmonary symptoms and lymphomatoid papulosis resolution Symptomatic rebound within days requiring imatinib Reduction in IL-5 level and eosinophilic count	NR
Wagner, 2009 (29)	750 mg Q4W	No positive effect associated with mepolizumab Increase in eosinophil levels	NR
Mehr, 2009 (30)	10 mg/Kg Q4W	Marked decrease in eosinophil counts and mean daily prednisolone after 3 mepolizumab infusions Complete clinical remission (skin rash and respiratory symptoms) until 3 months after third infusion	No adverse events
Schwartz, 2010 (41)	Dosage NR Q8W or Q12W	Dramatic improvement in quality of life Complete remission of gastrointestinal symptoms, peripheral oedema, erythema and seizure activity after 2 weeks of treatment Marked improvement of dyspnea and exercise tolerance	No adverse events attributable to mepolizumab
Kersey-Barrett, 2012 (32)	750 mg Q4W	Moderate improvement in erythroderma but requiring cyclosporine and eventual addition of prednisone to obtain complete control Decrease in eosinophilic count from 6.74 to 0.06×10^9^/L for 2 months	NR
Bleeker, 2012 (33)	750 mg Q4W	Reduction in absolute eosinophilic count to <0.2×10^9^/L in the 6 months since starting mepolizumab Excellent control of symptoms	Good mepolizumab tolerance Non-toxicity related to mepolizumab
D’Elbée, 2013 (34)	Dosage NR QW	After the first mepolizumab dose: Clinical worsening (mouth necrosis) Reduction in eosinophil count from 21 to 2.4×10^9^/L	NR
Roufosse, 2015 (35)	750 mg Q4W	Clear-cut regression of angioedema Decrease in eosinophil count levels	Lymphoid infiltration extended into the surrounding tissues (sixth mepolizumab infusion)
Klion, 2015 (36)	750 mg Q6W	Clinical improvement Reduction in eosinophilia count from 0.2 to 0.45×10^9^/L Discontinuation of glucocorticoid therapy	NR
Patel, 2016 (37)	750 mg Q4W	Normalization of eosinophilic count (0.03×10^9^/L) Reduction in regimen of systemic glucocorticoids At 6-month follow-up (mepolizumab therapy in combination with infliximab): Resolution of gastrointestinal symptoms Marked improvement in quality of life	Repeated Clostridium difficile infection requiring multiple courses of antibiotic therapy and resulting in ulcerative colitis Idiosyncratic drug hypersensitivity reaction to azathioprine
Song, 2017 (38)	100 mg Q4W	At 7-month follow-up: Reduction in prednisone dose to 15 mg/day Clinical stabilization	Good tolerance No adverse effects
Brunet, 2018 (39)	100 mg Q4W	Resolution of neurologic symptoms	NR
Matucci, 2018 (40)	750 mg Q4W	At 5-years follow-up: Reduction in annual rate and severity of exacerbations (10 ± 1.4 vs 2 ± 0.45, p <0.005) Reduction in eosinophilic count (2.737 vs 782; p <0.005)	No adverse events
Schwarz, 2018 (41)	750 mg Q4W or Q6W	Improvement in lung function and eosinophil blood count	No serious adverse events
Mulvey, 2018 (42)	100 mg Q4W	At 2-weeks follow-up: Reduction in eosinophilic count to 0.0×10^9^/L Symptoms resolution (fatigue and atopy)	NR
Klion, 2018 (15)	750 mg Q4W	Partial suppression of hypereosinophilia	Debilitating side effects for 3 to 5 days after each mepolizumab dose (fatigue, malaise, nausea, and vomiting)
To, 2018 (43)	100 mg Q4W	At 1-month follow-up: Resolution of symptoms Reduction in eosinophil counts to the normal range	No adverse events potentially attributed to mepolizumab
Eng, 2020 (44)	300 mg, interval dosing NR	Increase in eosinophil counts from 1.15 to 2.04×10^9^/L	NR
Domany, 2020 (45)	NR	Normalization of the eosinophil counts to 0.1×10^9^/L Successful tapering of glucocorticoids	NR
Kurosawa, 2020 (46)	100 mg Q4W	At 1-month follow-up: Decrease in eosinophil count to 0.044×10^9^/L Discontinuation of oral methylprednisolone Improvement of pulmonary function	No adverse effects associated with mepolizumab
Weiss, 2021 (47))	100 mg Q4W	Rapid improvement of cutaneous manifestations and lab values	NR
Helbig, 2021 (8)	300 mg Q4W	Significant resolution of clinical symptoms despite persistent melanoderma Normalization of eosinophilia No further glucocorticoid therapy	NR
Di Nora, 2021 (48)	100 mg Q4W	After treating a severe clinical deterioration to cardiogenic shock: Marked clinical improvement Reduction of peripheral hypereosinophilia	NR
Jonakowski, 2021 (49))	300 mg increased to 500 mg (September 2019) and 700 mg (December 2019) Q4W	Reduction in oral methylprednisolone dosage to 4 mg/d Improvement of neurologic symptoms (sensory and spasticity)	Good tolerance No evident adverse events
Eubanks, 2022 (50)	100 mg Q4W	At 14 days: Progressive reduction in absolute eosinophilic count to 3.37×10^9^/L Symptom resolution (fevers, fatigue and lymphadenopathies)	NR
Cascio, 2022 (51)	100 mg Q4W	At 1-week follow-up: Reduction in absolute eosinophilic count to 0.01×10^9^/L Glucocorticoids discontinuation with sustained response	No adverse effects
Jue, 2022 (52)	100 mg Q4W	Initial reduction below 0.1–0.2×10^9^/L with subsequent gradual increase leading to mepolizumab suspension	NR

IL-5, Interleukin-5; NR, Not reported; Q2W, every 2 weeks; Q4W, every 4 weeks; Q6W, every 6 weeks; Q8W, every 8 weeks; Q12W, every 12 weeks.

#### 3.4.1 Symptom remission and clinical response

Thirty of 35 (85.7%) studies (2 prospective, 5 retrospective, and 23 case reports including 93 patients) reported the impact of mepolizumab on symptom remission and clinical response. Two studies defined complete response as a symptomatic improvement and a decrease of the eosinophil count to within the normal range (0–0.5×10^9^/L) (3, 9), while another also used criteria such as concomitant treatment with ≤10 mg prednisone (21).

Data on mepolizumab remission rates were only available from a prospective study (24), in which five patients (71.4%) achieved clinical remission, including four with respiratory involvement and one with chronic rhinitis. Among the four retrospective series (n=56) with data on clinical response (9, 21, 24, 25), the complete response rate varied from 57.1% (with mepolizumab monotherapy) to 76.0% during a 1–6-month observation period. During this period, a partial response to mepolizumab 750 mg was also observed, with rates ranging from 11% (3) to 100% (9). A partial response was defined as a decrease in eosinophil count, but not necessarily in the normal range, and/or symptomatic improvement and/or requiring >10 mg prednisone and/or additional HES therapy. In the retrospective study by Kuang et al., non-responders (i.e., no symptomatic improvement after one month of mepolizumab and with a stable or increasing eosinophil count) comprised 8 of 35 patients (23%) (21). The baseline characteristics of non-responders to mepolizumab therapy included a median (range) duration of 6.13 (0.82–15.40) years for HES, a median peak absolute eosinophil count of 13.04 (5.40–79.00) × 10^9^/L, involvement of 4 (3–5) organ systems, and group IV of glucocorticoid sensitivity (i.e., unresponsive to 60 mg prednisone daily for ≥1 week) (21). Among the case reports, 11 (47.5%) and 10 (43.7%) patients reported complete and partial responses to mepolizumab, respectively. Most complete responses (mainly in patients with L-HES) were characterized by an improvement in baseline clinical manifestations, including a reduction in skin symptoms (rash and pruritus) and respiratory symptoms (cough and wheeze) in 45.4% and 36.6% of patients, respectively, as well as a reduction in gastrointestinal and neurological symptoms, among others. A lack of response to mepolizumab was observed in only two cases: one patient treated with a 100 mg dose for an unreported HES subtype (48) and another patient with unreported doses for L-HES (34).

The duration of remission varied between studies. In the two prospective series, Maule et al. reported sustained remission at 12 months, despite a reduction of glucocorticoids (23) while Paolini et al. observed variability in the duration of response (4–16 weeks; mean 10.2 weeks) with a median of 26-months follow-up period (range 7–52) (24). In one retrospective series (21), subjects who received >6 doses of mepolizumab reported improvement in therapy-related morbidity and significantly fewer disease flares (p<0.05) compared with subjects receiving conventional therapy.

Two studies investigated the existence of specific subgroups of patients with different responses to mepolizumab (9, 21). In the retrospective series from Carpentier et al., the clinical response was disappointing (9); while two patients experienced partial improvement of angioedema, the symptoms of associated muscle involvement were unchanged, and two patients experienced progressive worsening of cutaneous manifestations with persistent tissue eosinophilia (**Table 2**). In another retrospective series by Kuang et al., complete and partial response rates were higher among subjects who had enrolled after a clinical response was achieved in a prior mepolizumab trial (10 out of 12; 83%) than those who had enrolled based on treatment-refractory, life-threatening HES (10 out of 23; 43%) (21). Also, response to mepolizumab was more likely in subjects responsive to glucocorticoids with idiopathic or overlap forms of HES. An important benefit of mepolizumab treatment was the reduction of comorbidities observed after discontinuation or reduction of conventional HES therapies.

#### 3.4.2 Blood eosinophil counts

Thirty-one studies (2 prospective (23, 24), 2 retrospective (21, 26) and 27 case reports) reported the impact of mepolizumab treatment on blood eosinophil counts. For the 80 patients with HES included in these studies, baseline eosinophil counts ranged from 0.85 to 83.3×10^9^/L. Most studies (n=27, 77.1%) reported lower mean blood eosinophil counts following mepolizumab treatment (71.4–99.1% reductions from baseline), with post-treatment absolute counts ranging from 0.03–3.37×10^9^/L. Among the prospective series, Paolini et al. reported that one out of three patients (33.3%) experienced a decrease in blood eosinophil counts (24), and Maule et al. found that median eosinophil count decreased significantly within three months, reaching a median eosinophil count of 0.04×10^9^/L (IQR 700, p=0.008) at 12 months (23). Reductions in blood eosinophil counts were also observed in the two retrospective series, with counts returning to normal in 57% of patients but remaining elevated in 14.2% of partial responders (21) and 20% of non-responders (26). Of the 27 case reports, 24 (88.8%) indicated reductions in eosinophil count following treatment with mepolizumab; in 8 of these cases (29.6%), the blood eosinophil count returned to the normal range. Percentage reductions from baseline to follow-up in blood eosinophil counts ranged from 71.4% (40) to 99.1% among the case reports (32). Only 3 cases (11.1%) reported elevations in blood eosinophil counts following mepolizumab therapy (29, 44, 52); these included one patient with both HES and episodic angioedema with eosinophilia who had an initial decrease in blood eosinophil count prior to an elevation (52), and one patient who developed angioimmunoblastic T cell lymphoma (AITL) and may have had undetected AITL before mepolizumab (44). The third patient may have had eosinophilia that was not IL-5 independent or could have been producing excessive IL-5 that outpaced the effectiveness of mepolizumab injections (29).

#### 3.4.3 Glucocorticoid maintenance dosage

Fourteen studies (one prospective (23), two retrospective (9, 26), and 11 case reports (8, 30, 31, 36–38, 40, 45, 51, 52)) including 27 patients, reported data on the sparing effect of mepolizumab on glucocorticoid dose in patients with HES.

In 14 patients across five studies, the mean daily glucocorticoid dose ranged from 2–80 mg/day at mepolizumab initiation and was reduced to 3.5–15 mg/day over 3–60 months of follow-up after mepolizumab treatment (23, 26, 30, 38, 40). In the prospective study by Maule et al., reductions in prednisone dose from baseline were observed up to a median value of 5 mg/day (IQR: 6.25, p= 0.02) at 3 months (23). In the retrospective study from Benjamin et al., glucocorticoid dose was reduced from 13.8 mg/day at baseline to 3.5 mg/day at 6-month follow-up (p=0.13) (26). Similarly, seven case reports showed a 70–75% reduction in daily glucocorticoid dose from baseline across different periods (ranging from 2 weeks to 60 months) following treatment with mepolizumab.

Glucocorticoid discontinuation rates following mepolizumab therapy were reported in eight studies (one prospective (23), one retrospective (9) and six case reports (8, 27, 31, 36, 51, 52)), including a total of 18 patients with HES. Overall, glucocorticoid discontinuation rates ranged from 40–100% over a 1-week–2-year follow-up period. In the prospective study from Maule et al., 85.7% of patients (6/7) had discontinued glucocorticoids after 12 months of follow-up (23). In the retrospective study by Carpentier et al., 40% of patients receiving glucocorticoids at baseline had discontinued after 22 months of mepolizumab treatment (9). Among the case reports with available follow-up data, 6 patients (22.2%) discontinued glucocorticoid treatment between 1 week and 2 years of mepolizumab treatment.

#### 3.4.4 Other clinical outcomes

Mepolizumab treatment of patients with HES has been linked to improvements in quality of life (QoL) and symptoms of fatigue. Improvements in QoL were reported for two patients from one study who received unknown doses of mepolizumab; however, it was not stated whether the authors employed a validated scale to assess QoL in this study (41). Two patients in the study reported improvements in fatigue, including one who was able to complete a full day at his office after treatment with mepolizumab 750 mg (41). Similarly, in a case report by Mulvey et al., symptoms of fatigue had resolved in a patient with HES two weeks after a second 100 mg mepolizumab monthly injection (42). Other case studies have reported regression of fatigue three weeks after initiation of mepolizumab 750 mg (27), as well as the resolution of fever, fatigue, and lymphadenopathy after treatment with mepolizumab 100 mg (50).

### 3.5 Mepolizumab: safety

Twenty-one studies (two prospective (23, 24), two retrospectives (3, 21) and fourteen case reports) involving 81 patients reported data on mepolizumab safety. Overall, mepolizumab was considered safe and well tolerated in patients with HES; 34.3% of studies (two prospective with 7 patients each (23, 24) and ten case reports (27, 30, 33, 38, 40, 41, 43, 46, 49, 51)) did not report any adverse events during follow-up (n= 27 patients). A retrospective series by Kuang et al. reported malignancies in 4 of 23 patients (17.4%) treated with mepolizumab, including cases of basal cell carcinoma, colon cancer, angioimmunoblastic T cell lymphoma, and squamous cell carcinoma (21). However, the investigators did not explicitly link these findings to mepolizumab. Six patients (17.1%) discontinued mepolizumab therapy, including four due to malignancy, one who died, and one through patient choice (21). In another retrospective study, 10 patients (34%) discontinued anti-IL-5 treatment, including reslizumab, because of lack of efficacy, and 1 patient discontinued because of medication intolerance (3.4%) (3). Among the case reports, only five studies involving a total of 5 patients, reported adverse events after mepolizumab treatment, including the following isolated cases: generalized lymphadenopathy and itching (35); repeated Clostridium difficile infection that required multiple courses of treatment (37); debilitating side effects (fatigue, malaise, nausea, and vomiting) lasting for 3 to 5 days after each dose (15); angioimmunoblastic T cell lymphoma (AITL) with secondary large B cell proliferation, and cutaneous and lymphomatous involvement (AITL may have been present but undetected at the beginning of treatment) (44); and an acute flare of an HES episode that occurred during the weaning of glucocorticoids, defined by clinical features consistent with HES and an eosinophil count >1.5×10^9^/L (30). One case report described the discontinuation of mepolizumab treatment due to a progressive increase in eosinophil count despite continued injections of mepolizumab 100 mg every 4 weeks (this dosage <300 mg every 4 weeks is approved by both the FDA and EMA based on results of mepolizumab efficacy) (52).

Two retrospective studies (3, 21) and three case reports (34, 35, 45) provided data on mortality during mepolizumab treatment. Among the retrospective series, 2 (5.7%) patients died during the 5-year follow-up periods in the Kuang et al. series (21) and 4 patients (26.6%) died in the Ogbogu et al. series (3) had died. Two of the 4 deaths in the Ogbogu et al. series were considered secondary to HES (eosinophilic heart disease); the remaining deaths were not explicitly associated with mepolizumab treatment. Across three other case reports with available data, all 3 patients died during follow-up after treatment with mepolizumab; causes of death included acute respiratory failure and general sepsis (34), infection complications (35), and subarachnoid hemorrhage (44). However, the authors did not attribute the deaths to mepolizumab therapy, and no other safety events were reported.

### 3.6 Mepolizumab: effects in L-HES and M-HES populations

In 26 patients with L-HES, two retrospective series (3, 9) and six case reports (34, 35, 41, 44, 51, 52) showed remission and complete response rates of 76–100% following mepolizumab therapy; only one case study reported a non-responder to mepolizumab treatment (this patient had a progressing and painful general lingual enlargement requiring a tracheotomy and IFN-alpha treatment) (34). Four case studies reported a reduction and normalization (up to 88.6%) in blood eosinophil counts in 4 patients with L-HES (34, 35, 41, 51). In contrast, two studies showed an increase in eosinophil blood levels after treatment with mepolizumab 100 mg (one patient) and 300 mg (one patient), leading to discontinuation in both patients (44, 52). Only two case reports focused on patients with M-HES, with inconsistent results (29, 39). One case achieved a complete response to mepolizumab, with the resolution of neurologic symptoms and full recovery, alongside a reduction in eosinophil blood counts (39). In the second case study, eosinophil counts increased with 3-monthly infusions of mepolizumab 750 mg (29).

## 4 Discussion

Evidence generation is always a challenge in rare diseases such as HES. The inclusion of real-world studies with a high degree of external validity is of the greatest significance in enhancing the efficacy findings derived from RCTs within the current therapy landscape for patients with HES. Our review summarizes the available evidence regarding the effectiveness and safety of mepolizumab among patients with HES treated in a real-world clinical context. Treatment with mepolizumab has shown positive outcomes in patients with HES in a clinical setting. Up to three quarters of patients experienced improvements in the signs and symptoms of HES, and their eosinophil counts were reduced by 71.4–99.1% from baseline levels. This treatment also had a substantial glucocorticoid-sparing effect, with up to 85.7% of patients being able to discontinue the use of glucocorticoids after 12 months completely. The published real-world evidence reviewed showed that mepolizumab was generally safe and well tolerated, with no adverse events reported during follow-up in 12 of 35 studies (34.3%, including 27 patients).

The findings from the large number of patients included in our review indicate that the clinical advantages identified with mepolizumab in clinical trials regarding various subtypes of HES (53, 54) were also present in the real-world population, particularly in L-HES. The safety profile of mepolizumab is encouraging, even when considering its use for conditions beyond HES. While HES studies provide valuable insights, the real strength resides in the consistency of safety data observed in other, more established indications such as eosinophilic granulomatosis with polyangiitis, severe eosinophilic asthma, and nasal polyposis. Since studies of these other indications have notably more extensive data (larger number of patients and longer follow-up periods) in comparison to HES studies, they provide a more robust understanding of the mepolizumab safety profile in a broader context (55, 56).

The positive results we observed in real-world settings are consistent with previous controlled trials that assessed the use of mepolizumab in patients with HES (53, 54, 57, 58). In our comprehensive review, we found that mepolizumab treatment led to a substantial decrease of up to 99.1% in blood eosinophil count from baseline levels, which is in line with earlier clinical studies of mepolizumab in patients with HES. This noteworthy decrease in blood eosinophil count is important as it suggests a potential reduction in tissue eosinophilia and organ damage in individuals with HES on mepolizumab. Based on our findings, it seems that mepolizumab could be beneficial for patients with uncontrolled HES and might lead to a reduced need for additional treatments such as glucocorticoid, cytotoxic, or immunosuppressive therapy. However, we found various disparities among the real-world populations analyzed in this scoping review, where populations varied substantially from those included in RCTs in terms of disease severity, clinical manifestations, and concurrent medications.

Other authors have reviewed the existing body of literature regarding the efficacy and safety of mepolizumab in patients with HES in order to generate clinical practice recommendations (59). In the pivotal phase 3 RCT, the proportion of patients with HES experiencing at least one flare or withdrawing from the study was 50% lower with mepolizumab versus placebo (28% vs 56%; p=0.002) (53). Furthermore, mean blood eosinophil count was markedly reduced at Week 2 (170 cells/µL) in patients receiving mepolizumab compared with baseline (1460 cells/µL), and by Week 32 there was a 92% reduction in blood eosinophil count with mepolizumab versus placebo. Additionally, the proportion of patients with on-treatment adverse events was similar in the mepolizumab (89%) and placebo groups (87%) (53). Efficacy and safety of mepolizumab were maintained in the open-label extension study (54). The annualized flare rate in the previous placebo and mepolizumab groups was 0.37 and 0.14 events/year, respectively. In addition, mepolizumab reduced blood eosinophil count by 89% in patients previously receiving placebo and maintained a reduced blood eosinophil count in those previously treated with mepolizumab. Of patients receiving oral corticosteroids in weeks 0–4, 28% achieved a 50% or greater reduction in mean daily dose during weeks 16–20. There were no new safety signals identified in the open-label extension study (54).

There are several limitations that must be considered when interpreting the findings of this review. First, we included non-randomized samples from case reports and small series with incomplete data. Unlike mepolizumab for treating severe eosinophilic asthma, which has received extensive investigation (53, 54, 57, 58), few high-quality trials in patients with HES are available (60) due to the low frequency of the disease. To address this limitation, we performed an extensive search across two prominent databases, Ovid Medline and EMBASE, which identified 105 participants who received mepolizumab for HES. To our knowledge, this is the largest HES study of mepolizumab. Additionally, we adhered to the standards outlined by the PRISMA statement and employed rigorous selection criteria to ensure studies of sufficient methodological quality. Most of the studies (80%) involving mepolizumab in patients with HES were conducted using individual case reports, which do not allow for an in-depth evaluation of the topic and may restrict the interpretation of the findings. Second, a limited number of patients were included in the studies and were generally affiliated with the same group of authors. Due to the low incidence of HES and evolving features of L-HES, a unique case may be reported more than once at different stages of the illness, and duplicated inclusion of such a case cannot be entirely avoided. To mitigate this factor, we tried to combine populations from the same study to avoid duplicated information. Third, there is potential for publication bias, with favorable outcomes more likely to be reported. Finally, we observed considerable heterogeneity in study designs, inclusion criteria, HES subtypes, disease severity, mepolizumab dosing regimens, geographic locations (including regions where mepolizumab is not authorized), outcome definitions and follow-up across the included studies. Notably, outcome definitions for remission, partial response, and glucocorticoid sparing varied widely across studies. Delineating differences between certain HES variants may be challenging, and there are no reliable predictive markers of disease course or validated disease activity/remission measures in HES. In addition, the current diagnostic criteria and response treatment for HES are not uniform, and a definition needs to be posted more precisely. However, despite these limitations, the overall consistency of the obtained data and experimental evidence suggests that the results were not confounded. The findings of this study provide a comprehensive overview of the existing evidence, which consistently supports the favorable clinical outcomes associated with the use of mepolizumab in patients with HES.

There are several unresolved questions regarding the safety and effectiveness of mepolizumab in treating the diverse subtypes of HES. The optimal dosing strategy for mepolizumab in HES is yet to be determined. However, evidence from several studies supports the use of 300 mg mepolizumab SC every four weeks as the standard regimen (53, 54). In the phase 3 trial, mepolizumab (300 mg SC every four weeks) provided a significant reduction in disease flares compared with placebo (28% vs. 56%, respectively), with no additional safety concerns (53). Patients continued to show reduced flare rates, eosinophil counts and glucocorticoid dependence during the open-label extension study (54). Although early data suggest potential efficacy of lower doses of mepolizumab in idiopathic HES (61), the evidence is not yet sufficient to justify their routine use, and further research is required. There is also a lack of long-term real-world follow-up data beyond 1–2 years to assess potential predictors of response to mepolizumab, including HES subtype, peripheral blood eosinophil count, serum IL-5 and glucocorticoid sensitivity (21, 62).

The precise role of eosinophils as the principal mediators of disease manifestations in different subtypes of HES and the importance of IL-5 in these diverse disorders remain to be delineated. Furthermore, therapy development is also limited by HES assessment, as there are no validated disease activity measures to assess treatment response in patients with HES. This lack of information complicates the task of identifying treatment response, as well as the prediction of the likelihood of relapse during treatment ta2pering. The existence of well-defined clinical standards regarding the diagnosis, management, and treatment of patients is the highest priority since they will allow an adequate assessment of the use of mepolizumab at different dosages and routes of administration for treating HES. Future research should focus on the development of a variety of validated disease activity and patient-reported outcome measures to better characterize symptoms and mepolizumab effects, assisting in the formulation of consensus remission criteria in clinical practice and improving the management of patients with HES (63).

## 5 Conclusion

Mepolizumab provides symptom remission, decreases blood eosinophil counts, and demonstrates a significant glucocorticoid-sparing effect in patients with HES; it also has a favorable safety profile characterized by few and minor adverse events. This scoping review provides evidence that is linked to significant therapeutic benefits in individuals with HES receiving mepolizumab therapy in real-world clinical settings. Our findings support the evidence provided by RCTs regarding the effectiveness and safety of mepolizumab in HES and hence contribute to the development of future therapeutic strategies in this context.
