# Supplementary material for: Mepolizumab for hypereosinophilic syndrome: effectiveness and safety from real-world evidence
Source: Front Immunol. 2025 Nov 14;16:1704077. doi: 10.3389/fimmu.2025.1704077 (PMC12660071; doi:10.3389/fimmu.2025.1704077)
Supplement: Supplementary file 1 [file Table1.docx]

# Supplementary material

## Supplementary methods

### Search strategy for Ovid MEDLINE(R) <1946 to May Week 2 2023>

1 exp Hypereosinophilic Syndrome/ 5204

2 hypereosinophilic syndrom*.mp. 2849

3 Eosinophilic Leukemi*.mp. 548

4 Idiopathic Hypereosinophilic Syndrom*.mp. 568

5 Loeffler* Endocarditis.mp. 106

6 Lympho* variant hypereosinophilic syndrom*.mp. 29

7 Lympho* eosinophilia.mp. 42

8 Lympho* hypereosinophilia.mp. 3

9 Pulmonary Eosinophilia.mp. 3595

10 LHES.mp. 47

11 L-HES.mp. 39

12 HES-L.mp. 10

13 HES.mp. 6813

14 or/1-13 12808

15 Mepolizumab.mp. or mepolizumab/ 1011

16 Nucala.mp. 21

17 Bosatria.mp. 2

18 (SB-240563 or SB240563).af. 9

19 or/15-18 1014

20 Epidemiologic studies/ 9319

21 exp case control studies/ 1412574

22 exp cohort studies/ 2476846

23 Case control.tw. 133897

24 (cohort adj (study or studies)).tw. 265707

25 Cohort analy$.tw. 10020

26 (Follow up adj (study or studies)).tw. 51249

27 (observational adj (study or studies)).tw. 130285

28 Longitudinal.tw. 270377

29 Retrospective.tw. 617559

30 Cross sectional.tw. 411455

31 Cross-sectional studies/ 465032

32 (Real-World or Realworld).mp. 54304

33 (Real-Life or reallife).mp. 21498

34 case report/ or case report*.mp. 2190730

35 or/20-34 5566169

36 14 and 19 and 35 76

### Embase session results (19 May 2023)

#1 'hypereosinophilic syndrome'/exp OR 'hypereosinophilic syndrome' 4514

#2 'hypereosinophilic syndrom*':ti,ab,de,tn 4496

#3 'eosinophilic leukemi*':ti,ab,de,tn 1143

#4 'idiopathic hypereosinophilic syndrom*':ti,ab,de,tn 825

#5 'loeffler* endocarditis':ti,ab,de,tn 214

#6 'lympho* variant hypereosinophilic syndrom*':ti,ab,de,tn 51

#7 'lympho* eosinophilia':ti,ab,de,tn 83

#8 'lympho* hypereosinophilia':ti,ab,de,tn 6

#9 'hypereosinophilic syndrome'/exp OR 'hypereosinophilic syndrome' 4514

#10 'hypereosinophilic syndrom*':ti,ab,de,tn 4496

#11 'eosinophilic leukemi*':ti,ab,de,tn 1143

#12 'idiopathic hypereosinophilic syndrom*':ti,ab,de,tn 825

#13 'loeffler* endocarditis':ti,ab,de,tn 214

#14 'lympho* variant hypereosinophilic syndrom*':ti,ab,de,tn 51

#15 'lympho* eosinophilia':ti,ab,de,tn 83

#16 'lympho* hypereosinophilia':ti,ab,de,tn 6

#17 'pulmonary eosinophilia':ti,ab,de,tn 1110

#18 'lhes':ti,ab,de,tn 103

#19 'l-hes':ti,ab,de,tn 70

#20 'hes-l':ti,ab,de,tn 24

#21 'hes':ti,ab,de,tn 13020

#22 #9 OR #10 OR #11 OR #12 OR #13 OR #14 OR #15 OR #16 OR #17 OR #18 OR #19 OR #20 OR #21 18433

#23 mepolizumab OR 'mepolizumab'/de 4714

#24 nucala 147

#25 bosatria 36

#26 'sb 240563' OR sb240563 91

#27 #23 OR #24 OR #25 OR #26 4714

#28 'clinical study'/de 162893

#29 'case control study' 251109

#30 'family study'/de 26288

#31 'longitudinal study'/de 190609

#32 'hypereosinophilic syndrome'/exp OR 'hypereosinophilic syndrome' 4514

#33 'hypereosinophilic syndrom*':ti,ab,de,tn 4496

#34 'eosinophilic leukemi*':ti,ab,de,tn 1143

#35 'idiopathic hypereosinophilic syndrom*':ti,ab,de,tn 825

#36 'loeffler* endocarditis':ti,ab,de,tn 214

#37 'lympho* variant hypereosinophilic syndrom*':ti,ab,de,tn 51

#38 'lympho* eosinophilia':ti,ab,de,tn 83

#39 'lympho* hypereosinophilia':ti,ab,de,tn 6

#40 'pulmonary eosinophilia':ti,ab,de,tn 1110

#41 'lhes':ti,ab,de,tn 103

#42 'l-hes':ti,ab,de,tn 70

#43 'hes-l':ti,ab,de,tn 24

#44 'hes':ti,ab,de,tn 13020

#45 #32 OR #33 OR #34 OR #35 OR #36 OR #37 OR #38 OR #39 OR #40 OR #41 OR #42 OR #43 OR #44 18433

#46 mepolizumab OR 'mepolizumab'/de 4714

#47 nucala 147

#48 bosatria 36

#49 'sb 240563' OR sb240563 91

#50 #46 OR #47 OR #48 OR #49 4714

#51 'clinical study'/de 162893

#52 'case control study' 251109

#53 'family study'/de 26288

#54 'longitudinal study'/de 190609

#55 'retrospective study'/de 1426472

#56 'prospective study'/de 849701

#57 'randomized controlled trials'/de 251755

#58 #56 NOT #57 839461

#59 'cohort analysis'/de 991371

#60 cohort NEXT/1 (study OR studies) 457360

#61 ('case control' NEXT/1 (study OR studies)):ti,ab 164209

#62 ('follow up' NEXT/1 (study OR studies)):ti,ab 74891

#63 (observational NEXT/1 (study OR studies)):ti,ab 244743

#64 (epidemiologic? NEXT/1 (study OR studies)):ti,ab 7

#65 ('cross sectional' NEXT/1 (study OR studies)):ti,ab 328650

#66 #51 OR #52 OR #53 OR #54 OR #55 OR #56 OR #57 OR #58 OR #59 OR #60 OR #61 OR #62 OR #63 OR #64 OR #65 3953937

#67 'real world' OR realworld 131264

#68 'real life' OR reallife 47623

#69 'case report'/de OR 'case report$' 3052327

#70 #66 OR #67 OR #68 OR #69 7007829

#71 #45 AND #50 AND #70 195

#72 #71 AND [embase]/lim NOT ([embase]/lim AND [medline]/lim) 104

#73 #71 NOT #72 92
